# A Review on Low-Dose Emission Tomography Post-Reconstruction Denoising with Neural Network Approaches

**Published:** 2024-01-15

**Authors:** Alexandre Bousse, Venkata Sai Sundar Kandarpa, Kuangyu Shi, Kuang Gong, Jae Sung Lee, Chi Liu, Dimitris Visvikis

**Affiliations:** Univ. Brest, LATIM, INSERM UMR 1101, 29238 Brest, France.; Univ. Brest, LATIM, INSERM UMR 1101, 29238 Brest, France.; Lab for Artificial Intelligence & Translational Theranostics, Dept. Nuclear Medicine, Inselspital, University of Bern, 3010 Bern, Switzerland.; The Center for Advanced Medical Computing and Analysis, Massachusetts General Hospital/Harvard Medical School, Boston, MA 02114, USA; Department of Nuclear Medicine, Seoul National University College of Medicine, Seoul 03080, Korea; Department of Radiology and Biomedical Imaging, Yale University, New Haven, CT, USA; Univ. Brest, LATIM, INSERM UMR 1101, 29238 Brest, France.

**Keywords:** Low-Dose, PET, SPECT, Deep Learning

## Abstract

Low-dose emission tomography (ET) plays a crucial role in medical imaging, enabling the acquisition of functional information for various biological processes while minimizing the patient dose. However, the inherent randomness in the photon counting process is a source of noise which is amplified in low-dose ET. This review article provides an overview of existing post-processing techniques, with an emphasis on deep neural network (NN) approaches. Furthermore, we explore future directions in the field of NN-based low-dose ET. This comprehensive examination sheds light on the potential of deep learning in enhancing the quality and resolution of low-dose ET images, ultimately advancing the field of medical imaging.

## I. Introduction

The main components of ET are positron emission tomography (PET) and single-photon emission computed tomography (SPECT). They measure the radio-tracer distribution administered to the patient via gamma radiation arising from radioactive decay and have multiple use cases including oncology, cardiology, neurology, etc. The ability to get functional information on the various biological processes distinguish them from other imaging modalities such as MR imaging (MRI) and computed tomography (CT).

Radioactive decay is a random process which entails the difficulty in precise production of the images in ET. Noise or the speckled variation in ET images is caused by the inherent randomness of the photon counting process. In order to ensure patient safety, research has been extensively conducted in the regime of low-dose ET imaging [1], [2]. The reduction in the dose administered to the patient further adds to the challenge of obtaining a clear image.

ET images are reconstructed from the measured gamma rays, which is an ill-posed inverse problem subject to noise amplification. The images suffer from partial volume effects due to the low intrinsic resolution of the imaging systems, as well as positron range for PET. The resolution of the reconstructed images can be improved with model-based iterative reconstruction (MBIR) that incorporate the point spread function (PSF) in the system matrix. However, this further contributes to the ill-posedness of the inverse problem, which results in more noise. Hence, a number of post-processing techniques have been proposed in this regard. Artificial intelligence (AI) and more specifically deep learning-based methods (i.e., deep NNs) have been very effective in denoising and super resolution and hence have been explored at length in ET. The focus of this article is to discuss at length the NN approaches that have been proposed for image denoising in low-dose ET.

**Fig. 1.**
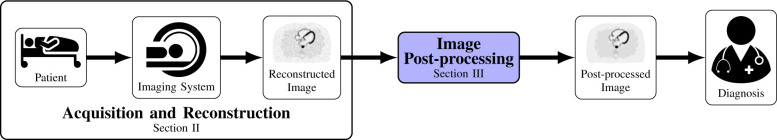
Main steps in clinical ET. The patient is initially administered with a radioactive tracer. The emission is captured by an imaging system. The mapping of the raw detector data to an image is performed by an image reconstruction method. Finally, after post-processing, the image is used for diagnosis.

The main steps in the clinical application of ET, are highlighted in Fig. 1. The first step is the production of the radio-pharmaceutical followed by its administration to the patient. Following this the emission data is acquired by a detector setup. Using the physical forward model, image reconstruction methods are utilized to map the raw detector data to an image. These steps are summarized in Section II. Following reconstruction, the image can be post-processed to reduce the noise and improve resolution. Section III gives an overview of existing post-processing techniques for low-dose ET image post-processing, with an emphasis on NN-based approaches, and is the main contribution of this paper. Finally, Section IV covers future directions of NN-based low-dose ET.

## II. Data Acquisition and Image Reconstruction

### A. Data Acquisition

SPECT imaging is based on the emission of a single gamma photon per each radioactive decay event. These gamma photons are usually collected by a gamma camera [3], which typically consists of a collimator that selects relevant gamma photons to be detected; the gamma photons are converted into light in the visible spectra by the scintillation crystal. The optical-wavelength photons are sent to a photomultiplier tube (PMT) that converts light into electrons, generating a detectable current. This current is then measured by an electronics setup, noting the occurrence of the event. The relative spatial coordinates of the event are determined by measurements from the point of contact in the PMTs. These events are stored in histograms based on their position, resulting in discrete (or vectorized) projection measurement data which is then utilized for image reconstruction.

PET on the other hand uses positron emitting radioisotope. The positron interacts with an electron resulting in an annihilation event that produces two gamma photons moving in nearly opposite direction. These gamma photons are simultaneously detected (coincidence event) by circularly arranged detector elements. Due to this inherent feature of PET, collimators are not present in the detector system. The coordinates of each decay event are recorded, through the detection of the corresponding coinciding pair of gamma photons. The binning of these detected coincidence events results in the projection data, used for reconstruction.

ET image reconstruction and post-processing requires a formalism that we briefly describe here. The radiotracer distribution takes the form of an image vector $x={\left[x_{1},\ldots ,x_{m}\right]}^{⊤}\in R^{m},\;‘⊤’$, denoting the matrix transpositioon, where each entry $x_{j}$ denotes the radiotracer concentration at voxel j (in Bq per voxel). In both SPECT and PET, the imaging system is modeled by a system matrix $P\in R^{n\times m}$ where for all (i,j) each entry $[P{]}_{i,j}$ is the probability that an emission in voxel j is detected along the ith line of response (LOR), taking into account the geometry of the system, the linear attenuation, the sensitivity of the detectors and the intrinsic resolution. For each LOR i=1,…,n, the expected number of detections given a radiotracer distribution x is

(1) $${\overset{‾}{y}}_{i}(x)=\tau [Px{]}_{i}+r_{i}$$

where τ is the scan duration and $r_{i}$ is a background term comprising expected scatter as well as randoms (for PET), and the number of detection is a random variable $y_{i}$ that follows a Poisson distribution centered in $\overset{-}{y}(x)$, i.e.,

(2) $$y_{i}∼Poisson\left({\overset{‾}{y}}_{i}(x)\right).$$

In the following we denote $y={\left[y_{1},\ldots ,y_{n}\right]}^{⊤}$ and $\overset{-}{y}(x)={\left[{\overset{‾}{y}}_{1}(x),\ldots ,{\overset{‾}{y}}_{n}(x)\right]}^{⊤}$ the measured and expected data respectively, and $r={\left[r_{1},\ldots ,r_{n}\right]}^{⊤}$ the background events vector.

### B. Reconstruction

Reconstructing an image $x^{\text{rec}}$ corresponds to solving the following inverse problem:

(3) $$\text{finding}\;x^{\text{rec}\;}\text{s.t.}\;y\approx \overset{-}{y}(x^{\text{rec}}),$$

This can be achieved by analytical inversion of P applied to $\frac{1}{\tau }(y-r)$, also known as filtered-backprojection (FBP) (see for example [4]). Unfortunately, the inverse problem (3) is ill-posed and direct inversion leads to noise amplification which is impractical for low-dose imaging. Moreover, the inversion relies on an idealized model that does not incorporate resolution modeling. Finally, solving (3) does not guarantee positivity of the solution.

Another approach consists in finding an estimate $x^{\text{rec}}$ by penalized maximum log-likelihood (PML)

(4) $$x^{\text{rec}}\in \underset{x\in R_{+}^{m}}{argmin}\;\ell (y,\overset{-}{y}(x))+\beta R(x)$$

where $\ell (y,\overset{-}{y})$ is the Poisson negative log-likelihood of the expectation $\overset{-}{y}$ given the measurement y,R is a penalty, or *prior*, that enforces image smoothness and β>0 is a weight. Solving (4) can be achieved by MBIR such as the expectation-maximization (EM) algorithm [5] or ordered-subset EM (OSEM) algorithm [6] in absence of penalty (i.e., β=0), and modified EM [7] with a smooth convex penalty. Reconstructing the image x by PML (4) reduces the noise as compared with analytical inversion (3) by (i) the incorporation of the stochastic model of the noise in L and (ii) by the presence of the penalty that controls the noise. Typical penalty term R includes quadratic smoothness penalty [8], the edge-preserving Huber penalty [9] or the relative difference prior [10]. Anatomical priors have been used to smooth the PET image while preserving image resolution by taking advantage of high-resolution anatomical images such as CT or MRI [11]–[15]. More recently, Sudarshan *et al.* [16], [17] introduced patch-based dictionary learning for joint PET/magnetic resonance (MR) image reconstruction.

PML methods have played a pivotal role in ET image reconstruction. These methods have proven effective in managing noise levels and improving image quality. However, in the context of low-dose ET imaging, striking a suitable balance between noise reduction and preservation of essential image details necessitates careful tuning of the prior weight. Failure to do so may lead to undesired over-smoothing artifacts. Furthermore, anatomically-guided penalties, while valuable in certain cases, can introduce artifacts when there is misalignment between the activity and anatomical images. It is also worth noting that the applicability of PML techniques is contingent upon the availability of raw data, which may not always be obtainable.

## III. Deep Learning-based Image Post-processing

When ET raw data are not available, images can be post-processed to improve their quality. Prior to the advent of deep learning, conventional image post-processing methods were employed for incorporating corrections in ET images. The first image post-processing task is to improve the image resolution, namely, partial volume correction (PVC). PVC first achieved with deconvolution techniques, which consists of correcting for the image PSF using iterative techniques, such as the Van Citert algorithm [18] or the Richardson-Lucy algorithm [19], [20]. However, deconvolution is an ill-posed inverse problem and leads to noise amplification, which is non-practical for low-dose imaging. Therefore, it is necessary to deploy adequate techniques to control the noise. This topic has been the subject to numerous works over the last decade [21]–[25].

The inherent relationship between resolution and noise poses a significant challenge. Enhancing image resolution typically leads to increased noise levels, while reducing noise tends to compromise resolution. Addressing both aspects simultaneously requires a paradigm shift towards AI-based approaches. NNs have revolutionized imaging ever since the performance of AlexNet in the ImageNet challenge [26]. In medical imaging, they have contributed to image segmentation, cancer detection, registration, reconstruction, etc. [27]. In the context of low-dose ET imaging, they have been widely implemented to bring about improvements in images reconstructed by traditional algorithms.

This section reviews the NN-based low-dose ET denoising methods and categorizing them based on their NN design. We have broadly categorized the methods into supervised methods, self-attention (SA) mechanisms, unsupervised methods, multi-modality (i.e., additional anatomical information from another modality) and diffusion models (DMs). These subsections have further been divided into subcategories to distinguish and highlight specific workings of the approaches. Note that, although SA mechanisms are typically categorized as supervised methods, recognizing their increase in popularity and effectiveness, we have chosen to dedicate a separate section to these mechanisms, with an emphasis on transformers.

### A. Supervised Methods

Machine learning methods that require labeled data for training come under this category. Owing to the data revolution and the availability of annotated datasets for different tasks along with competitions, the most popular methods for denoising are supervised.

A reconstructed image $x^{\text{rec}}$ produced by traditional methods like FBP (by solving (3)) or PML (by solving (4)) is processed through an image-to-image NN $F_{\theta }$ depending of a trained parameter $\theta^{⋆}$ as

(5) $$x^{pp}=F_{\theta ⋆}\;\left(x^{rec}\right)$$

where $x^{pp}$ is the post-processed image. Supervised training of $\theta^{⋆}$ is generally achieved using a training dataset of K noisy/clean image pairs $\left(x_{k}^{\text{noisy}},x_{k}^{\text{clean}}\right),\;k=1,\ldots ,K$, as

(6) $$\theta^{⋆}=\underset{\theta }{\text{arg}\;\text{min}}\sum_{k=1}^{K}L\left(F_{\theta }\left(x_{k}^{\text{noisy}\;}\right),x_{k}^{\text{clean}\;}\right)$$

where L(·,·) is a loss function.

There are many possible variations of the NN $F_{\theta }$ and its training. This subsection will cover fully convolutional networks (FCNs) and generative adversarial networks (GANs).

#### 1) Fully Convolutional Networks:

**Fig. 2.**
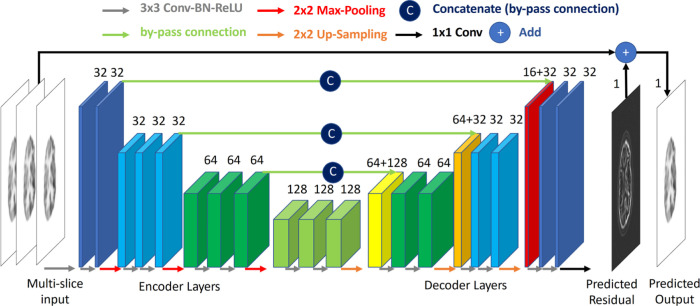
Representation of supervised deep learning-based method from Xu *et al.* [30].

Computer vision tasks like image segmentation, super-resolution, image enhancement, etc., typically utilize FCNs that are based on residual neural network (ResNet) [28] or U-Net [29]. One of the earliest works that used FCNs for denoising in PET was proposed in [30]. The proposed method predicted full-dose PET images from PET images with dosage reduced by 200 times. The authors used a convolutional encoder-decoder-styled architecture with three convolutional layers on the encoder and decoder part of the network. The encoder consisted of convolutions and max-pooling layers while the decoder consisted of upsampling through bilinear interpolation. In order to tackle the resolution loss experienced in the encoder-decoder type of structure the authors also employed concatenations between the encoder and decoder similar to a U-Net. In addition, a residual connection from the input to the output image was used. This enabled the network to learn the difference between the full-dose and low-dose PET images. The network was also trained with multi-slice input so as to help the network distinguish between noise and finer structural details. The loss function of choice in this work was the *L*^1^-norm. This approach is shown in Fig. 2. Gong *et al.* [31] proposed an architecture based on ResNet consisting of five residual blocks. Owing to the limited amount of real data, the authors initially trained the network on simulated data created using BrainWeb [32] and the extended cardiac-torso (XCAT) phantom [33]. The network was then fine-tuned on real data. Another aspect of this work is the use of perceptual loss to further improve the quality of predicted images. The authors found their proposed method to perform better than traditional Gaussian filtering. Dilated convolution [34] replaced the convolution operation in the U-Net in the work proposed by Spuhler *et al.* [35]. The network, called dNet, outperformed U-Net for PET image denoising. The advantage of dilated convolutions, which were first introduced for image segmentation, is that they remove the requirement of pooling and upsampling operators. The dataset used in this study was from a psychiatric study consisting of 35 patients. Deep denoising autoencoder (DAE) was proposed in [36] for dynamic PET denoising. The DAE was trained on noisy and noiseless spatiotemporal patches of simulated images. Although a promising voxel-level denoising method was proposed, the proposed DAE struggled to generalize to test data different from the training data. A 3-dimensional (3-D) version of the U-Net was used in [37] for mapping from noisy 64×64×64 patches to noise-reduced patches of the same dimension. The authors trained the network on a lung cancer real dataset. The method was evaluated by three physicians through lesion detection tasks. The proposed method performed better than Gaussian smoothing, but its improvements were limited when it came to the count levels typically observed in a clinical setting. The effect on noise levels for denoising PET images was studied in [38]. A personalized weighting strategy for specific noise levels was proposed through the linear blending of results from different models. The authors trained five 3-D U-Nets each with a different noise level in the training data. Along with these, a separate network with all the varied noise levels also was trained. The one-network-for-all model did not generalize well on the testing data with multiple noise levels. The networks trained on noisier images performed better at denoising but introduced more spatial blurring. The final method fused the deep image prior and regularization by denoising (RED) approach to obtain a final denoised image. DeepRED optimization was done using alternating direction method of multipliers [39].

Low-dose myocardial perfusion (MP) SPECT with deep learning was proposed in [40], where the authors used a 3-D convolutional neural network (CNN). The 3-D network consisted of autoencoders where the encoder and the decoder parts of the network were stacked with convolutions. The network was trained to map from various levels of dosage (1/2, 1/4, ..., 1/16) to full-dose images. The authors found their method to perform better than the conventional spatial-post filtering method. The network was trained on real patient data reconstructed with both FBP and OSEM algorithms. A four-layer U-Net was utilized in [41] for SPECT denoising. The U-Net in this method was trained on simulated XCAT phantoms. Low-dose imaging in SPECT through the reduction in acquisition time and projection angles was explored in [42]. The authors used a ResNet for mapping from low-dose to full-dose images. The dataset consisted of MP SPECT images of 363 patients recontructed with OSEM algorithm. A study on weighted loss functions for varying levels of statistics based on inter-subject changes for deep learning-based SPECT denoising was proposed in [43].

#### 2) Generative Adversarial Networks:

GANs [44] are a special type of NN model consisting of two units, with the generator unit synthesizing candidates while the discriminator unit attempts to decipher whether the candidate’s images are synthetic or real. The development of GANs has strengthened the capability of NNs in this regard, allowing them to capture complex probability distributions. Lu *et al.* [45] investigated the accuracy of deep learning-based denoising methods including GANs for PET imaging of small lung nodules, focusing on quantitative accuracy and visual image quality. Ouyang *et al.* [46] explored GANs with feature matching and task-specific perceptual loss in the restoration of amyloid PET. Jeong *et al.* [47] demonstrated that GAN-based restoration of amyloid PET scans did not affect physician interpretation, indicating that the restored images were consistent with the original scans and preserved their diagnostic value.

Conditional GAN (cGAN) was introduced by Wang *et al.* [48] to recover full-dose brain [^18^F]FDG PET images from low-dose measurements. Xue *et al.* [49] confirmed the cross-scanner and cross-tracer capability of customized cGAN, where the training was done from [^18^F]FDG PET on one scanner and the test was performed on [^18^F]FET and [^18^F]Florbetapir PET imaging of different scanners.

Cycle-consistent GAN (CycleGAN) was applied to recover full-dose whole-body (WB) PET from low-dose measurements [50]. Zhou *et al.* [51] confirmed that their CycleGAN preserves edges and standardized uptake values from the restored low-dose dataset with biopsy-proven primary lung cancer or suspicious radiological abnormalities. A supervised GAN with the cycle-consistency loss, Wasserstein distance loss, and an additional supervised learning loss, named as S-CycleGAN was demonstrated to outperformed 3-D-cGAN in the recovery of low-dose brain PET [52]. The CycleGAN has demonstrated the advantage to train non-synthetic low-dose WB [^18^F]FDG PET scans together with separate full-dose WB [^18^F]FDG PET scans in a study by Sanaat *et al.* [53].

**Fig. 3.**
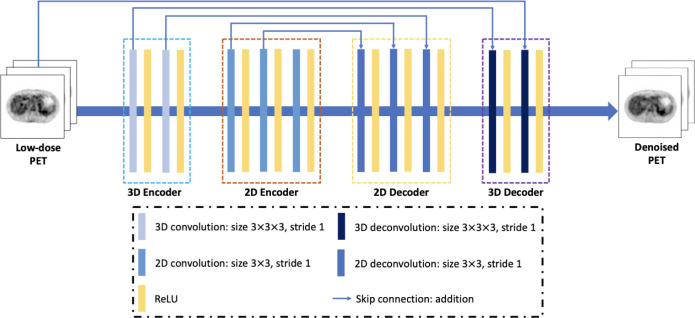
Structure of PT-WGAN from Gong *et al.* [54].

Gong *et al.* [54] proposed a parameter-transferred Wasserstein GAN (W-GAN), namely, PT-GAN, with a task-specific initialization for low-dose PET image denoising without compromising structural details. A representation of PT-GAN is shown in Fig. 3. Du *et al.* [55] developed a cascaded data consistency GAN to recover high-quality PET images from FBP-reconstructed PET images with streaking artifacts and high noise. Geng *et al.* [56] developed a content-noise complementary learning pipeline using GAN to reduce noise in medical images including CT, MR, and PET, which outperformed state-of-the-art denoising algorithms in terms of visual quality, quantitative metrics and robust generalization capability.

Additional modality such as MRI can be added into the training of GAN. Wang *et al.* [57] developed a 3-D auto-context-based locality-adaptive multi-modality GAN (LAGAN) for estimating full-dose [^18^F]FDG PET images from low-dose counterparts together with MR images and demonstrated the superiority over traditional multi-modality fusion methods for PET restoration. Zhou *et al.* [58] introduced a unified motion correction and denoising GAN for generating motion-compensated low-noise images from low-dose gated PET data.

GANs can be also applied on denoising directly during the reconstruction from low-count sinogram data. Xue *et al.* [59] use a LCPR-Net to enforced a cyclic consistency constraint on the least-squares loss of a GAN framework to establish a nonlinear end-to-end mapping process from low-count sinograms to full-count PET images. Similarly, an improved W-GAN framework was employed as a direct PET image reconstruction network (DPIR-Net) to enhance image speed and quality of PET reconstruction [60].

GANs were also applied on the denoising of SPECT images. Sun *et al.* [61] developed a method based on a 3-D cGAN for denoising of dual-gated MP images. The same group also investigated the denoising performance of cGAN in projection-domain and compared it with the denoising in reconstruction-domain for low-dose MP SPECT imaging [62]. Sun *et al.* [63] introduced Pix2Pix GAN for denoising low-dose MP images and found it superior than other denoising methods. They also introduced attention mechanisms in GANs for the denoising of fast MP images [64]. Aghakhan Olia *et al.* [65] developed a GAN to predict non-gated standard-dose SPECT images in the projection space. Their finding revealed that recovery of underlying signals/information in low-dose images beyond a quarter of the standard dose would not be feasible and adversely affect the clinical interpretation of the resulting images.

### B. Self-attention Mechanisms

The SA mechanism was first proposed for natural language processing by Vaswani *et al.* [66]. The generated attention map is similar to the weight elements utilized in the nonlocal means (NLM) denoising [67]. This connection was further explained in the work of nonlocal NNs[68].

During SA calculation, features are first extracted from the input to construct the *Query*, *Key*, and *Value* components. The *Query* and *Key* components are then utilized to generate the attention map through a matrix multiplication, scaling, and SoftMax operations. The calculated attention map is multiplied with the *Value* component to obtain the final output. Compared to the widely used convolution operation, the SA module has a spatially-variant filter defined by the attention map, which is calculated from the input itself. The SA module can be embedded into the popular U-Net and GAN structures to further improve the performance as demonstrated in various computer vision tasks [69], [70].

For PET image denoising, Xue *et al.* [71] proposed embedding the SA block into the widely used U-Net structure. The SA can also be embedded into other network structures for PET image denoising, such as GAN [72] and CycleGAN [73], [74]. Apart from utilizing only low-dose PET images as the input, Onishi *et al.* [75] proposed an unsupervised PET image denoising framework that incorporated anatomical information into the network architecture via the SA mechanism. The attention gates employed in this work aimed to remove PET image noise by better utilizing the multi-scale semantic features extracted from the MR prior image. For dynamic PET imaging, Li *et al.* [76] proposed to directly generate high-quality Patlak images from five-frame sinograms by a network with SA blocks, potentially reducing the acquisition time and avoiding the input function needed for parametric imaging. For PET image reconstruction, Xie *et al.* [77] proposed to utilize the U-Net with a SA block for image representation and further employ this network representation into the PET MBIR framework. They also employed additional high-resolution anatomical images as the network input to further improve the reconstruction performance [78].

Though CNNs achieved great success in various medical imaging tasks, the network specifically focused on local spatial information, and the receptive field was also limited. The transformer networks extensively employed SA blocks (described in the previous subsection) as the network building blocks, which had the ability to capture long-range information. Vision transformer (ViT) [79] was the first application of the transformer network to computer vision. The input images were firstly divided into patches, linearly embedded along with the position information, and then fed to the transformer network for image classification. One issue of ViT was the quadratically growing computational complexity along the spatial dimension, which limited the receptive field achievable.

**Fig. 4.**
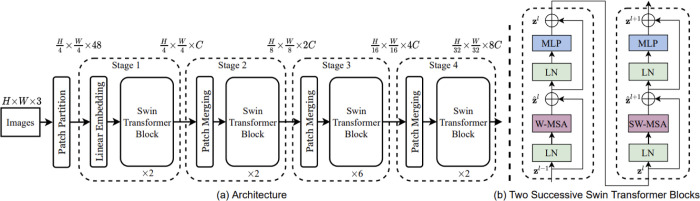
(a) The architecture of the Swin transformer network and (b) consecutive blocks of the Swin transformer. Reprint from Liu *et al.* [80].

To address this issue, the Swin Transformer [80] was proposed to efficiently calculate local multi-head self-attention (MSA) using shifted windows that offered linear computational complexity. Compared to ViT, the Swin Transformer divided the whole image into windows. The SA was computed based on a shifted windowing scheme, which achieved greater efficiency by limiting SA calculation to non-overlapping local windows while also considering cross-window connections. This method is represented in Fig. 4. Instead of SA calculation along the spatial domain, Restormer [81] was recently proposed to efficiently compute global MSA along the channel dimension. It had linear computational complexity for image restoration tasks.

The transformer networks were recently applied for PET image quality improvement. Luo *et al.* [82] proposed a GAN embedded with a transformer to perform low-dose PET image denoising. The transformer was inserted between the encoder and decoder paths of the generator network, and the training function was based on both voxel-wise estimation error and the adversarial loss. Jang *et al.* [83] proposed a transformer network that can leverage both spatial and channel information based on local and global MSAs. Quantitative evaluations based on datasets of different PET tracers, i.e., [^18^F]FDG, [^18^F]ACBC, [^18^F]DCFPyL, and [^68^Ga]DOTATATE, showed that the proposed transformer structure achieved better performance than other reference methods. When utilizing both low-dose PET and high-resolution MR prior images as the input, Zhang *et al.* [84] designed a network structure with two paths to extract PET and MR features and a transformer block to fuse the PET and MR features. Wang *et al.* [85] compared five deep learning-based denoising methods, including Swin Transformer and ViT, under different PET dose levels. Results showed that the Swin Transformer achieved better performance than other reference methods in most evaluation tasks. Apart from PET image denoising, Hu *et al.* [86] utilized the transformer network for PET image reconstruction following the unrolled-NN framework [87]–[89], and the results showed better performance than other unrolled networks where CNNs were adopted.

### C. Unsupervised Methods

The advent of unsupervised methods was a result of the limitations that supervised learning methods posed namely large datasets and the effort that goes into annotating them. Two such methods that have been developed in the context of denoising are deep image prior (DIP) and Noise2Noise (N2N) methods.

#### 1) Deep Image Prior:

The DIP [90] is a learning-free method that demonstrated that a randomly initialized NN could be used as a prior for inverse problems like denoising, inpainting, and super-resolution. The DIP changes the paradigm of the standard deep denoising approach (5) in the sense that instead of training the parameter θ from a dataset as described in (6), the post-processed image $x^{pp}$ is obtained from the reconstructed image $x^{\text{rec}}$ as

(7) $$\theta^{⋆}=\underset{\theta }{arg\;min\;}L\left(F_{\theta }(z),x^{rec}\right),\;x^{pp}=F_{\theta^{⋆}}(z),$$

where z is a random image input. This approach relies on the implicit regularization imposed by the architecture of the NN $F_{\theta }$ which prevents over-fitting, the structure of a NN being sufficient to capture the low-level information of the image. This section describes the denoising methods in ET that utilize DIP.

A 3-D U-Net was trained in [91], [92] to predict denoised PET images using CT/MRI images as the high-quality prior input (i.e., z in (7)) and noisy PET images as the label. It was observed that using a prior image further improved the results rather than training the NN on a random noise input (as done in [90]). The network was trained on a single set of MR images and noisy PET volumes and then evaluated on simulated phantoms as well as real datasets. The loss function used was the mean squared error (MSE) combined with the limited-memory Broyden-Fletcher-Goldfarb-Shanno algorithm [93]. The DIP method was extended to dynamic PET denoising in [94]. The 3-D U-Net in this work was trained on static PET images as inputs to the network and noisy dynamic PET images as training labels. DeepRED [95] is a method that utilized DIP with RED [96]. A U-Net-like architecture was trained on noisy dynamic PET images with MSE. The input to the network was a random noise vector. Hashimoto *et al.* extended their previous work [94] in [97] where they proposed two modular approaches to dynamic PET image denoising. The first module consisting of a 3-D U-Net extracted features from static PET images. The extracted features were then fed to a reconstruction module consisting of a typical CNN with convolution layers that predicted denoised images while being trained on noisy dynamic PET volumes. Simultaneous denoising of dynamic PET images was proposed by Yang *et al.* [98]. Their network consisted of multiple convolutional layers, taking as input time averaged PET images with the training labels being the noisy dynamic PET volume. A variation of this network called double DIP was also proposed which additionally generates the time averaged PET images.

#### 2) Noise2noise:

N2N is a self-supervised learning (SSL) technique, i.e., a machine learning technique that trains models using input data only, without labeled data and explicit supervision. Labeling data takes considerable time and effort and in some situation obtaining gold standard-labeled data is impossible. SSL has the advantage of significantly increasing the number of datasets for model training as it does not require labeled data. In medical imaging techniques that employ ionizing radiation, such as X-ray and gamma-ray, obtaining a clean target (label) with a high radiation dose can elevate the potential health risks associated with radiation exposure. Although it is possible to obtain a clean target by increasing the scanning time, it may cause image blurring or distortion by increasing the likelihood of patient movement.

The N2N approach, as proposed by Lehtinen *et al.* [99], trains a deep NN $F_{\theta }$ parametrized with θ for image denoising using K noisy image pairs, i.e., two noise instances of the same image, as input and target, as follows:

(8) $$\theta^{⋆}=\underset{\theta }{\text{arg}\;\text{min}}\sum_{k=1}^{K}{\left\|F_{\theta },\left(x_{k}^{\text{clean}\;}+\epsilon_{k,1}\right)-\left(x_{k}^{\text{clean}\;}+\epsilon_{k,2}\right)\right\|}^{2}$$

where $\epsilon_{k,1},\;\epsilon_{k,2}$ are independent noise realizations for each image $x_{k}^{\text{clean}},\;k=1,\ldots ,K$. This SSL technique, which only requires the input and target noise distribution to be identical and independent, has demonstrated its efficacy in reducing various types of noise, including Gaussian and Poisson noise.

There has been a growing interest in applying the N2N to medical images, such as CT and MRI [100]–[102]. In these studies, the denoising efficacy of N2N was compared to that of conventional denoising techniques. CT images reconstructed using N2N prior images yielded better root MSE and structural similarity index measure (SSIM), as well as improved texture preservation as compared with conventional methods using total variation, NLM, and convolutional sparse coding [100]. Fang *et al.* [101] showed that using a N2N-denoised image as a prior within MBIR showed promising results in suppressing noise while preserving subtle structures when applied to spectral CT data for material decomposition. Jung *et al.* [102] evaluated the performance of N2N for image quality improvement in sub-millimeter resolution 3-D MR images. In this study, the K-space data of 3-D MR images were split into two separate sets with independent noise realizations, which was achieved by undersampling data alternatively along the *k-z*-axis and estimating missing data using a GRAPPA kernel. Volumetric accuracy, as well as image quality, was improved by N2N, which utilized only a single fully sampled K-space data.

**Fig. 5.**
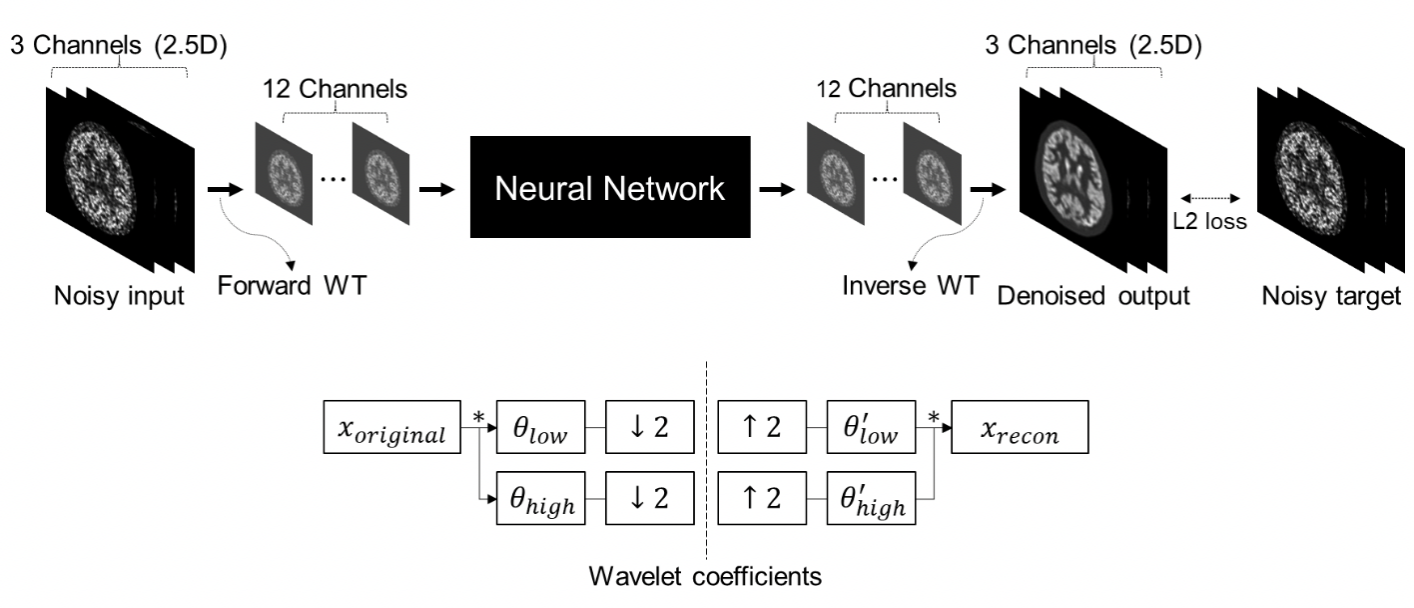
Schematic of N2N network model improved by the incorporation of WTs. Reprint from Kang *et al.* [107].

**Fig. 6.**
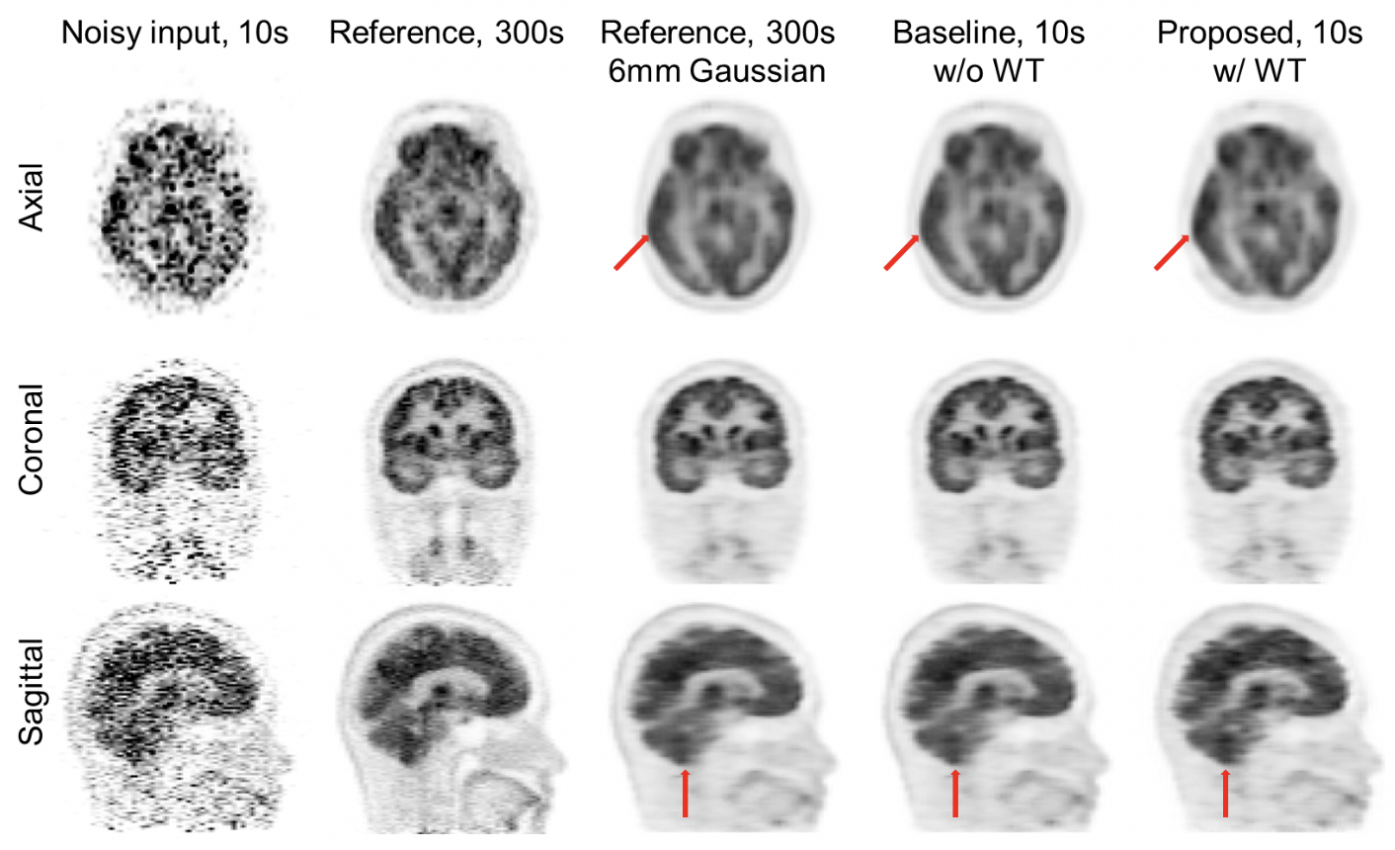
Full count image, noisy input, Gaussian filtered and denoised images using the N2N without and with incorporating the trainable WT for clinical data. Reprint from Kang *et al.* [107].

N2N and SSL technology are also potentially useful for improving the image quality and diagnostic performance in ET. List-mode data recorded in nuclear medicine image scans and containing detail information about the location, energy, and time of each detected event allows for flexible data binning. Its flexibility has provided excellent platform for investigating and implementing N2N technologies that require identical and independent dataset as input and target for network training. A N2N application study on [^18^F]FDG brain PET utilized short time-bin images (10s–40s) generated from list-mode data and showed equivalent peak signal-to-noise ratio of N2N outcomes compared to supervised learning with 300s images as target, at all tested noise levels [103]. The usefulness of N2N for noise reduction in [^15^O]water dynamic PET and [^99m^Tc]MDP/DPD WB bone scan studies was also reported [104], [105]. In addition, Chan *et al.* [106] proposed a technique to improve the accuracy and robustness of N2N network trained for WB PET image denoising by mitigating the high variance in N2N denoising outcomes. The variability is mainly caused by the spatially non-stationary nature of PET image noise distribution. In this study, instead of training the network with pairs of individual noisy realizations, the number of training samples was increased by pairing a single noisy realization with an ensemble of noisy realizations at the same count level. When applied to low-count WB PET images, the original N2N produced speckle and clustered noise artifacts. Nevertheless, the proposed method was effective in reducing the noise while preserving natural noise texture. An endeavor has been also undertaken to enhance the generalization ability of N2N-based noise reduction algorithm through the incorporation of wavelet transforms (WTs) into the N2N network [107]. The proposed method entails the utilization of the forward WT to decompose a given noisy image into its low-pass and high-pass frequency components. These data are then fed into an N2N network, followed by an inverse WT. The final output was compared with another noisy image as in typical N2N framework. The forward and backward WT coefficients were also determined through training, thereby enabling the proposed method to outperform the original N2N method in suppressing artifacts and preserving abnormal uptakes. This method is shown in Fig. 5 and Fig. 6.

Multiple variants of N2N have been developed to mitigate different types of noise and enhance the efficacy of self-supervised denoising algorithms. These variants include Noiser2Noise, Noise2Void, and Noise2Self [108]–[110]. When applied to low-count [^18^F]FDG brain PET images, Noiser2Noise, which requires only a single noisy realization of each training sample, was more effective than N2N in preserving the noise texture of the input images [103]. Noise2Void, another SSL technique that does not require paired training samples, outperformed traditional denoising methods for PET when pre-trained through transfer learning and guided by anatomical images [111].

### D. Multi-modality

In hybrid PET/CT, PET/MR, and SPECT/CT systems, the anatomical information derived from CT and MRI can also be used to enhance the denoising performance of PET and SPECT. Numerous studies using CNNs in brain PET/MR datasets achieved substantial dose reduction, sometimes up to 99%, and incremental improvements in image quality and resolution have been demonstrated by combining PET and MR images as multiple channels into the network compared to using PET images alone as input [57], [112]–[116]. While CNNs requirement on training datasets is high, a relatively low-complexity CNN (micro-net) that is more robust to very limited amounts of training data was proposed for MR-guided PET denoising, and demonstrated to have robust performance [117]. Other networks structures, such as a spatial adaptive and transformer fusion network [84], have also been proposed to improve PET denoising by more effectively incorporating MRI information.

Not only helping noise reduction, anatomical CT and/or MRI information can also improve the PET image resolution. For example, anatomically-guided PET reconstruction using the Bowsher prior [118] can be generated by a CNN in the image, thus space allowing the generation of anatomically-guided high-resolution PET images without the need to access raw data and reconstruction console [119]. When PET raw data are available, incorporating sinogram-based physics into the loss function of PET/MR networks has been shown to further improve the denoising performance that is more robust to outof-distribution (OOD) data [120].

In addition to the strategy of incorporating anatomical images as multi-channel inputs, such information can also be input to the network through a SA mechanism, for unsupervised PET denoising for example [75], [78].

In another strategy, investigations using MR and CT images as prior information to guide DIP (cf. Section III-C1) have been performed for brain and body PET/CT datasets [91], [92], [121]. The group further extended the anatomical-guided DIP approach to direct parametric reconstruction framework, where CT and MR images were incorporated as the network input to provide a manifold constraint, and also utilized to construct a kernel layer to perform non-local feature denoising [122].

One requirement of incorporating anatomical information into PET denoising is the integrated or simultaneous scanners such as PET/MR, which are still limited to widespread use. A study suggested that accurate full-dose amyloid PET images can be generated from low-dose PET and either simultaneous or non-simultaneous (acquired up to 42 days apart) MR images, broadening the utility of low-dose amyloid PET imaging [123].

While a large number of hybrid PET denoising work were performed for brain PET/MR datasets, investigations using CT and/or MRI information for denoising body PET images were also performed. In an application to cardiac viability [^18^F]FDG imaging in patients with ischemic heart disease, both attenuation correction CT and low-count PET were input into networks through two channels, leading to effective denoising [124]. In a study of [^68^Ga]PSMA prostate PET/MR, 50% dose reduction could be achieved by using a discrete-WT CNN with MRI priors [125]. Rather than concatenating the MR and PET at the input level, a study combined them in the feature space with attention-weighted loss, and applied the methods to WB PET for children and young adults lymphoma patients [126].

While brain MR images are usually well registered with PET images, for WB applications, mismatch between PET and CT/MR due to motion could complicate PET denoising methods that incorporate anatomical information. A study showed that when CT and PET are aligned, incorporating CT as additional channels improves the quantitative accuracy of lung lesions derived from denoised low-count PET images. However, when CT and PET are misaligned, incorporating CT information resulted in additional lesion quantification bias as compared with using PET data only [45]. The results suggest that motion correction and image registration are important pre-processing steps when incorporating anatomical information into PET denoising.

Similar approaches described above can also be applied to hybrid SPECT/CT scanners. In a SPECT bone imaging application, a lesion-attention weighted U^2^-Net incorporating both 1/7-count SPECT and resampled attenuation correction CT can lead to the successful generation of synthetic standard-count SPECT images [127].

### E. Diffusion Models

DMs have arisen as an alternative to GANs for image generation and other tasks [128], and have been widely explored in medical imaging [129]. In the following paragraph we briefly summarize score-based DMs for image generation as described in Ho *et al.* [130].

Assume that the (clean) feasible images x are distributed according to some unknown probability distribution function (PDF) $p_{0}(x)$ that we wish to use to randomly generate images. The forward diffusion is achieved by generating a collection of images ${\left(x_{t}\right)}_{t=1,\ldots ,T}$, starting from a clean image $x=x_{0}$ drawn from $p_{0}$ (i.e., by randomly choosing an image from the training dataset), as a Markov chain defined as

(9) $$x_{t}|x_{t-1}∼𝒩\left(\sqrt{\alpha_{t}}x_{t-1},\left(1-\alpha_{t}\right)I\right)$$

where I is the identity operator in the image space, and ${\left(\alpha_{t}\right)}_{t=1,\ldots ,T}$ is a collection of parameters in ]0, 1] defined such that $x_{T}$ is (approximately) a white noise. Sampling an image according to the initial distribution $p_{0}$can therefore be achieved by reversing the diffusion process, i.e., starting from a white noise $x_{T}$ from which $x_{T-1}$ is generated, and so on until $x_{0}$. Unfortunately, the reverse conditional PDFs $p\left(x_{t-1}|x_{t}\right)$ are unknown. Instead, the approximate model is used:

(10) $$x_{t-1}|x_{t}∼𝒩\left(\mu \left(x_{t},t\right),\sigma^{2}(t)I\right)$$

where $\sigma^{2}(t)$ is a known function of $\alpha_{t}$ and $\mu \left(x_{t},t\right)=\frac{1}{\sqrt{\alpha_{t}}}x_{t}+\frac{1-\alpha_{t}}{\sqrt{\alpha_{t}}}\nabla log\;p_{t}\left(x_{t}\right)$ where $p_{t}$ is the PDF of $x_{t}$. The score function $s_{t}\left(x_{t}\right)=\nabla log\;p_{t}\left(x_{t}\right)$ is untractable and is therefore replace by a $S_{\theta }\left(x_{t},t\right)$, where the parameter θ is trained unsupervisingly via score-matching from several instances of ${\left(x_{t}\right)}_{t=1,\ldots ,T}$ generated following (9) and by sampling $x_{0}$ from a training dataset of clean images [131].

In addition to generating images, DMs can also be used for inverse problems such as denoising and reconstruction. One type of approach, namely conditional diffusion models (CDMs), consists in generating a noise-free image x according to the a-posteriori $p_{0}(\cdot |y)$, where y is the measurement (in our case, $y=x^{\text{noisy}}$), by successive sampling of $x_{t-1}|x_{t},y$. This can be achieved using the same above-mentioned methodology using the conditional score $s_{t}\left(x_{t},y\right)=\nabla log\;p_{t}(\cdot |y)\left(x_{t}\right)$. The conditional score can be approximated score-matching in two ways: (i) by conditional score-matching of a NN $S_{\theta }\left(x_{t},y,t\right)$ that takes as input both the image $x_{t}$ for all t and the measurement y [128], [132], [133] (supervised), and (ii) unconditional score-matching of $S_{\theta }\left(x_{t},t\right)$ (cf. previous paragraph) combined with the Bayes rule and an approximation of the posterior distribution $p\left(y|x_{t}\right)$ [134], [135] (unsupervised). Note that approach (ii) requires the knowledge of the noise model of y. With a different formulation but in the same spirit, Mardani *et al.* [136] proposed to approximate the a-posteriori PDF $p_{0}(\cdot |y)$ by minimization of the Kullback-Leibler divergence between a standard Gaussian model and the posterior, which yields to a penalized least-squares optimization problem with a score-matching penalty.

Recently, Gong *et al.* [137] used a CDM for brain PET image denoising with incorporation of the MR image as prior information y to the approximated conditional score $S_{\theta }\left(x_{t},y,t\right)$ trained from a clean PET/MRI dataset. Pan *et al.* [138] used a CDM reinforced with a consistency model [139] to improve the efficiency. Shen *et al.* [140] proposed to utilize diffusion models for low-count PET image denoising through a bidirectional condition diffusion probabilistic model, which was validated on WB PET datasets. Jiang *et al.* [141] proposed an unsupervised PET enhancement framework through the latent diffusion model, which can be trained only on standard-count PET data. Instead of a Gaussian noise, a Poisson noise was inserted in the diffusion process to better accommodate PET imaging. Also, a CT-guided cross-attention was proposed to incorporate additional CT images into the inverse process. Han *et al.* [142] proposes to generate high-quality PET based on the diffusion models through a coarseto-fine PET reconstruction framework that consists of a coarse prediction module and an iterative refinement module.

Apart from PET image denoising, Xie *et al.* [143] proposed to generate synthetic PET image from MR images based on the diffusion models. Singh *et al.* [144] proposed a diffusion model-based PET image reconstruction framework, where the PET forward model (eq. (1) and eq. (2)) was utilized together with the score function (i.e., approach (ii) above with the PET raw data *y*) for high-quality PET image generation. Their method was further improved by adding MR anatomical prior.

Despite their potential in image denoising for ET, it is important to note that DMs are relatively novel in this field. As of the current literature review, there is a limited number of research papers that have explored the application of DMs to denoise (or reconstruct) ET images. This scarcity of studies underlines the emerging nature of DMs in ET image processing, and as a result, it represents an exciting and promising avenue for further research and development.

## IV. Discussion and Conclusion

Reduction in the dosage of the radio-pharmaceutical administered to a patient undergoing a functional imaging scan is essential to reduce the risk and radiation the patient is being exposed to. Low-dose imaging aims to produce images with quality on par with regular-dose imaging while operating on a fraction of the radio-pharmaceutical. Getting a clear image in ET imaging is a difficult task due to attenuation, scatter, and the ill-posed nature of the physics model used to characterize PET and SPECT. Low-dose ET further complicates an already challenging problem. Images are produced by converting the detector data into readable images through the process of reconstruction. Low-dose ET reconstructed images suffer from noise. One way to tackle this noise is through the post-processing of the reconstructed images.

Image processing methods like Gaussian filtering have been implemented over the years to tackle the reconstructed noisy images. Recently deep learning-based methods have been found to surpass traditional algorithms in imaging tasks. In this article, various post-processing approaches that utilize deep NNs have been discussed at length. These approaches have been appropriately classified into various sub-sections to facilitate better readability and to distinguish them from one another. U-Net was the earliest NN adapted for denoising. Adding a discriminator network to a U-Net resulted in GANs. These networks were discussed in the Section III-A. Recently developed supervised methods include a self-attention mechanism to U-Net and GANs were highlighted in Section III-B. Deep image prior and N2N methods are the most popular unsupervised NN approaches, and were discussed in Section III-C. Section III-D presents methods that utilize multi-modality input data for training NNs Finally, Section III-E covers recent trends in DMs. The primary focus of this article has been to discuss the diverse set of NN-based approaches while keeping up with the latest trends in the denoising regime. The presented papers are summarized in Table I.

One of the main challenges in training a NN is the availability of data. There is an increasing number of publicly available datasets such as Brainweb [32]. However, these are limited to the use cases they have been compiled for. There is a concern about using NNs trained on one specific dataset to be generalized to a larger data pool. This challenge of dealing with OOD data is addressed to an extent by unsupervised methods, which do not require large labeled datasets. However, unsupervised methods are yet to perform convincingly better than supervised methods. Efforts are being made in this regard to develop NNs that produce robust results independent of labeled data.

Evaluation of the denoised images predicted is another aspect that all NN methods need to be held accountable for. Typically, MSE and SSIM are used for quantitative comparison and analysis. Plot profiles and region of interest analysis for tumors are also utilized for a more thorough evaluation. Some articles have further employed a scoring system used by radiologists for the qualitative assessment of the images. Such efforts to check the images produced by NN-based approaches are essential to build trust and translate these methods to clinical cases.

NN-based denoising has indeed revolutionized low-dose ET imaging. This article through the emphasis on both PET and SPECT has highlighted the plethora of ways deep learning has improved image quality through post-processing. We discussed the earliest methods as well as the most promising methods in recent times that have the potential to be translated into clinical usage. The advantage of denoising is that methods applicable to one modality can easily be implemented for other modalities also, and this review on low-dose ET could be useful to image denoising and enhancement in other modalities too.

The necessity to compare the multitude of methods presented in this review poses a formidable challenge, primarily due to their application-specific nature, spanning cardiology, neurology, and oncology, as well as the utilization of diverse training datasets. In an ideal scenario, method performances would be evaluated using dedicated benchmark datasets specifically designed for comparative purposes, accompanied by standardized metrics akin to those employed in renowned challenges within the field. Drawing inspiration from successful model evaluation frameworks, such as the Ultra-low Dose PET Imaging Challenge [145], it is paramount for the community to consider the creation of specialized datasets and standardized metrics aimed at comprehensively evaluating these methods. Such initiatives hold the potential to significantly enhance our ability to objectively compare and contrast the efficacy of different techniques across diverse medical applications, thus enabling more informed decisions and driving progress within the field.

## Figures and Tables

**TABLE I:** Summary of the reviewed articles

Application	Supervised	Method/Architecture	Anatomical Information	References

Section III-A: Supervised Methods				
Brain PET	yes	CNN, U-Net	no	[30], [35]
Brain & Chest PET	yes	ResNet	no	[31]
Brain PET	yes	DAE	no	[36]
Chest PET	yes	U-Net	no	[37]
Whole-body PET	yes	U-Net	no	[38]
MP SPECT	yes	CNN, U-Net, ResNet	no	[40]-[43]
Chest PET	yes	GAN, cGAN, CycleGAN, W-GAN	no	[49], [51], [54], [56], [58], [59]
Chest PET	yes	CNN, U-Net, GAN	CT	[45]
Brain PET	yes	GAN	no	[46], [47]
Brain PET	yes	GAN, cGAN,CycleGAN	no	[48], [52], [55]
Brain PET	yes	cGAN	MRI	[57]
Whole-body PET	yes	CycleGAN	no	[50], [53], [60]
MP SPECT	yes	cGAN, attention-based GAN	no	[61]-[65]

Section III-B: SA Mechanisms				
Whole-body PET	yes	U-Net + SA block	no	[71]
Chest PET	yes	U-Net + SA block	no	[77]
Chest PET	yes	U-Net + SA block	CT, MRI	[78]
Brain PET	yes	GAN + SA block	no	[72]
WB PET	yes	CNN, CycleGAN + SA block	no	[73], [76]
Brain PET	no	CNN + SA block	MRI	[75]
WB PET	yes	Transformer	no	[82], [83]
Brain, WB PET	yes	U-Net, GAN, Transformer	MRI	[84], [85]
Brain PET	yes	Transformer	no	[86]

Section III-C: Unsupervised Methods				
WB PET	no	DIP	CT, MRI	[91], [92]
Brain PET	no	DIP	no	[94], [95], [97], [98]
Brain PET	no	N2N	no	[103], [107], [111]
Bone SPECT	no	N2N	no	[105]
^15^O water PET	no	N2N	no	[104]
WB PET	no	N2N	no	[106]

Section III-D: Multi-modality				
Brain PET	yes	CNN, U-Net	MRI	[112]-[117], [119], [120], [123]
Brain PET	no	DIP	MRI	[122]
^68^Ga PET (prostate)	yes	CNN	MRI	[125]
MP PET	yes	U-Net	CT	[124]
WB PET	yes	CNN	MRI	[126]
Bone SPECT	yes	U-Net	CT	[127]

Section III-E: DMs				
Brain PET	no	DM	MRI	[137]
WB PET	no	DM	no	[138], [140]
WB PET	no	DM	CT	[141]
Brain PET	yes	DM	no	[142]
Brain PET (image synthesis)	yes	DM	MRI	[143]
Brain PET (image reconstruction)	no	DM	MRI	[142]

## Acronyms

3-D: 3-dimensional
AI: artificial intelligence
CDM: conditional diffusion model
cGAN: conditional GAN
CNN: convolutional neural network
CT: computed tomography
CycleGAN: cycle-consistent GAN
DAE: deep denoising autoencoder
DIP: deep image prior
DM: diffusion model
EM: expectation-maximization
ET: emission tomography
FBP: filtered-backprojection
FCN: fully convolutional network
GAN: generative adversarial network
LOR: line of response
MBIR: model-based iterative reconstruction
MP: myocardial perfusion
MR: magnetic resonance
MRI: MR imaging
MSA: multi-head self-attention
MSE: mean squared error
N2N: Noise2Noise
NLM: nonlocal means
NN: neural network
OOD: out-of-distribution
OSEM: ordered-subset EM
PDF: probability distribution function
PET: positron emission tomography
PML: penalized maximum log-likelihood
PMT: photomultiplier tube
PSF: point spread function
PVC: partial volume correction
RED: regularization by denoising
ResNet: residual neural network
SA: self-attention
SPECT: single-photon emission computed tomography
SSIM: structural similarity index measure
SSL: self-supervised learning
ViT: vision transformer
W-GAN: Wasserstein GAN
WB: whole-body
WT: wavelet transform
XCAT: extended cardiac-torso
